# Prevalence, Risk Factors and Outcomes Associated with Acute Kidney Injury in Patients Hospitalized for COVID-19: A Comparative Study between Diabetic and Nondiabetic Patients

**DOI:** 10.1155/2021/6666086

**Published:** 2021-01-06

**Authors:** Shayesteh Khalili, Tahereh Sabaghian, Meghdad Sedaghat, Zahra Soroureddin, Elham Askari, Neda Khalili

**Affiliations:** ^1^Department of Internal Medicine, Imam Hossein Hospital, Shahid Beheshti University of Medical Sciences, Tehran, Iran; ^2^Department of Nephrology, Shahid Beheshti University of Medical Sciences, Tehran, Iran; ^3^Chronic Respiratory Diseases Research Center, National Research Institute of Tuberculosis and Lung Diseases (NRITLD), Shahid Beheshti University of Medical Sciences, Tehran, Iran; ^4^School of Medicine, Tehran University of Medical Sciences, Tehran, Iran

## Abstract

**Background:**

The risk factors for acute kidney injury (AKI) development in patients with diabetes hospitalized for COVID-19 have not been fully studied yet. In this study, we aimed to estimate the rate of AKI among the hospitalized population with COVID-19 and to identify the risk factors associated with AKI among patients with diabetes. *Material and Methods.* This retrospective cohort study included 254 patients (127 with diabetes and 127 without diabetes) who were admitted for COVID-19 to a tertiary hospital in Tehran, Iran, between February and May 2020. Clinical characteristics and outcomes, radiological findings, and laboratory data, including data on AKI, hematuria, and proteinuria were recorded and analyzed.

**Results:**

Of 254 patients, 142 (55.9%) were male and the mean (± SD) age was 65.7 years (±12.5). In total, 58 patients (22.8%) developed AKI during hospitalization, of whom 36 patients had diabetes (*p* = 0.04); most patients (74.1%) had stage 1 or 2 AKI. Also, 8 patients (13.8%) required renal replacement therapy (RRT) after developing AKI. Regardless of diabetes status, patients who developed AKI had significantly higher mortality rates compared with patients who did not develop AKI (*p* = 0.02). Hematuria and proteinuria were observed in 38.1% and 55% of patients, respectively. Multivariate analysis showed that invasive mechanical ventilation, proteinuria, HBA1c level, history of cardiovascular disease, and use of statins were independent risk factors for AKI development in patients with diabetes.

**Conclusion:**

Results of this study showed that AKI develops in a considerable percentage of patients with COVID-19, especially in those with diabetes, and is significantly associated with mortality.

## 1. Introduction

Based on the world health organization (WHO) situation report dated August 6, 2020, more than 18.6 million confirmed cases of coronavirus disease-2019 (COVID-19) and approximately 703000 deaths have been reported worldwide [1]. Patients infected with severe acute respiratory syndrome coronavirus-2 (SARS-CoV-2) mainly develop mild disease; however, about 5% of infected patients experience a more severe form of disease with acute respiratory distress syndrome (ARDS), septic shock, and multiple organ failure [2].

Kidney involvement is a common finding in patients hospitalized for COVID-19 with up to 75% of patients experiencing proteinuria, hematuria, and/or acute kidney injury (AKI) at some point during hospitalization [3, 4]. Despite not receiving much attention, AKI is one the most significant complications associated with COVID-19 [5]. Among risk factors, diabetes, the need for invasive mechanical ventilation, older age, and cardiovascular disease are shown to be associated with greater risk of AKI development [6–8].

Several contributory factors such as suppressed immune system and overproduction of inflammatory cytokines make patients with diabetes prone to bacterial and viral pulmonary infections [9, 10]. Initial reports from some of the hardest-hit countries during the COVID-19 pandemic indicated that older individuals, male patients, and those with underlying comorbidities are at increased risk for morbidity and mortality associated with COVID-19 pneumonia [11–13]. Among these subpopulations, the management of COVID-19 in patients with diabetes has been of significant importance, particularly considering the greater risk of AKI development and the caution needed for use of specific medications in these patients [14]. Identifying the potential risk factors associated with the development of AKI in patients with coexistent COVID-19 and diabetes could guide therapeutic strategies and subsequently improve the prognosis of such patients. Thus, this study is aimed at estimating the prevalence of AKI in patients hospitalized for COVID-19, to investigate whether patients with diabetes are at higher risk of developing AKI compared with patients without diabetes and to identify the risk factors for AKI development.

## 2. Material and Methods

### 2.1. Study Design and Participants

In this retrospective cohort study, we recruited a total of 254 patients who were admitted for COVID-19 to a tertiary hospital in Tehran, Iran, between February and May 2020. Final follow-up date was July 15^th^, 2020. The study cohort included 127 patients with diabetes mellitus type 2 (according to medical records and/or patients' statement) and 127 patients without diabetes. Diagnosis of COVID-19 pneumonia was made by either a positive real-time reverse transcription polymerase chain reaction (RT-PCR) assay or consistent clinical and radiological findings. Due to the unavailability of RT-PCR kits for detection of SARS-CoV-2 early in the outbreak in Iran, patients who had clinical features and chest computed tomography (CT) findings highly suggestive of COVID-19 were considered as positive cases and, thus, were included in this study. Individuals with diabetes mellitus type 1, history of kidney transplant, and end-stage renal disease and also those on dialysis were excluded from the study.

### 2.2. Study Outcomes and Variables

The main outcome of this study was AKI development. Definition and staging of AKI was based on the Kidney Disease: Improving Global Outcomes (KDIGO) guideline [15]. The value of serum creatinine at hospital admission was considered as baseline renal function. For all patients, demographic data (age, sex, and body mass index (BMI)), underlying comorbidities, presenting symptoms, respiratory rate, pulse rate, oxygen saturation (on room air) and blood pressure at admission, laboratory examinations, radiological findings, clinical outcomes, and COVID-19-related complications (septic shock and multiple organ failure) were collected and analyzed. Laboratory examinations consisted of the total count of leukocytes, lymphocytes and neutrophils, hemoglobin level, platelet count, lactate dehydrogenase (LDH), C-reactive protein (CRP), erythrocyte sedimentation rate (ESR), creatine phosphokinase (CPK), albumin, procalcitonin, D-dimer, serum creatinine, blood urea, glomerular filtration rate (GFR), aspartate aminotransferase (AST), and alanine aminotransferase (ALT). Results of urine tests (dipstick and microscopic examination of urine sediment) were collected when available for the assessment of proteinuria and hematuria. Hematuria was defined as the presence of three or more urine red blood cells per high-powered field after at least two urine tests were obtained within 24 hours apart. GFR was computed by using the modification of diet in renal disease (MDRD) equation [16]. Preexisting chronic kidney disease was identified using prior electronic medical records, laboratory data (defined as GFR < 60 mL/min/1.73 m^2^ and/or albuminuria of at least 3 months duration), and history taking. For patients with diabetes, serum hemoglobin A1c (HbA1c) level and duration of diabetes were also recorded. In those who developed AKI, serum creatinine levels were measured daily during hospitalization. Jaffe's kinetic method was used for creatinine measurement in this study. RT-PCR assay was performed on nasopharyngeal specimens for SARS-CoV-2 detection with TaqMan® Premix TAKARA (TaKaRa, Dalian, China) considering the protocols provided by the manufacturer. Chest CT severity score was defined by the summation of individual scores from 5 lung lobes: scores of 0, 1, 2, 3, 4, and 5 were, respectively, assigned for each lobe if pulmonary involvement was 0%, less than 5%, 5%-25%, 26%-49%, 50%-75%, or more than 75% of each region. The range of total severity score was from 0 (no involvement) to 25 (maximum involvement).

Written informed consent was obtained from the patients prior to study enrollment and the study was approved by the ethics committee of Shahid Beheshti University of Medical Sciences (Ethics ID: IR.SBMU.RETECH.REC.1399.063).

### 2.3. Statistical Analysis

Normally distributed and nonnormally distributed continuous variables were expressed as mean (±SD) and median (interquartile range (IQR)), respectively, and categorical variables were presented as frequency (percentage). Normal distribution was evaluated by using the Shapiro-Wilk test. Categorical data were compared by using the chi-square test or Fisher's exact test, and continuous data were analyzed using independent samples*t*-test or Mann–Whitney *U* test as appropriate. Logistic regression (backward stepwise method) was performed for assessing the covariates associated with AKI development after entering variables with *p* value < 0.3 into the model. For multivariate analysis, urine protein was considered a dichotomous variable (proteinuria of any degree and negative/trace proteinuria). All statistical analyses were performed by using STATA software version 14 (StataCorp, Texas, USA). *p* values < 0.05 were considered statistically significant.

## 3. Results

### 3.1. Clinical Characteristics and Laboratory Data

In this cohort, 254 patients hospitalized for COVID-19, including 127 patients with diabetes and 127 without diabetes, were enrolled. The mean (± SD) age of patients was 65.7 years (±12.5), and more men were admitted than women (55.9% vs 44.1%). In the diabetes group, patients had a mean age of 66.4 years (±12.5), while patients in the nondiabetes group had a mean age of 65.0 years (±12.5). Patients' demographic and clinical characteristics are demonstrated in Table 1.

**Figure 1:**
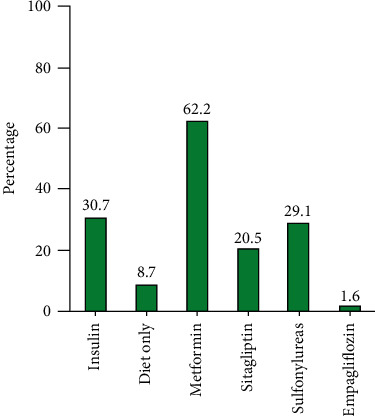
Medications used by patients for diabetes.

On admission, patients with diabetes had significantly higher respiratory rates and reduced oxygen saturation compared to patients without diabetes (*p* = 0.02 and *p* < 0.001); also, cough and dyspnea were more frequently seen in patients with diabetes (*p* = 0.04 and *p* = 0.05, respectively). Compared to patients without diabetes, significantly higher BMI levels were detected in patients with diabetes (*p* = 0.001). In terms of comorbidities, patients with diabetes had significantly higher rates of any underlying comorbidity (*p* < 0.001). History of hypertension, cerebrovascular disease, chronic kidney disease, and cardiovascular disease were also more common in patients with diabetes (*p* < 0.001, *p* = 0.01, *p* = 0.01, and *p* = 0.03, respectively). The medications that were used by study subjects during hospitalization are summarized in Table 2. As shown, heparin was the most commonly used medication among all patients (99.2%) followed by value="angiotensin-converting enzyme inhibitor/angiotensin II receptor blocker (ACEI/ARB)" (31.1%). In the group with diabetes, metformin and insulin were used by 62.2% and 30.7% of patients, respectively (Figure 1). More than 92% of patients received hydroxychloroquine as part of their treatment for COVID-19.

Patients with diabetes had significantly higher CT severity scores than patients without diabetes (*p* = 0.02). In total, 48 patients (18.9%) experienced death, 29 patients with diabetes, and 19 patients without diabetes (*p* = 0.11). Invasive mechanical ventilation was more common among patients with diabetes (*p* = 0.03), but complications including septic shock and multiple organ failure were not statistically different between the two groups (*p* = 0.18 and *p* = 0.20, respectively). The average length of hospitalization among all patients was 6 days (IQR, 3-10 days). Furthermore, patients without diabetes had a shorter duration of in-hospital stay in comparison to patients with diabetes (*p* < 0.001).

The median (IQR) serum creatinine levels at admission and discharge were 1.8 mg/dL (1.4-2.4) and 1.6 mg/dL (1.2-2.6), respectively. Among laboratory examinations, neutrophil counts and blood urea levels were significantly higher in patients with diabetes. In addition, patients with diabetes had marginally reduced hemoglobin and albumin levels and increased leukocyte counts compared to patients without diabetes (*p* = 0.05, *p* = 0.05, and *p* = 0.07, respectively). All other laboratory tests were not statistically different between the two groups (Table 1).

### 3.2. Kidney Involvement and Acute Kidney Injury

Among the study cohort, 58 patients (22.8%) developed AKI during hospitalization, of whom 36 patients had diabetes (*p* = 0.04); most patients (74.1%) had stage 1 or 2 AKI. Patients with diabetes had more severe AKI as opposed to those without diabetes (*p* = 0.03). In total, 8 patients (13.8%) required renal replacement therapy (RRT) after developing AKI. On urine tests, 45% of the patients had negative/trace protein, while the remaining patients (55%) experienced some degree of proteinuria. Moreover, 38.1% of the patients had hematuria on microscopic examination of urine sediment (Table 3). By univariate analysis, the following variables were significantly associated with AKI development in patients with diabetes: cardiovascular disease, use of statins, ACEI/ARB, and diuretics, invasive mechanical ventilation, CT severity score, HBA1c level, proteinuria and hematuria. After adjusting for potential confounders, invasive mechanical ventilation, proteinuria, HBA1c level, history of cardiovascular disease, and use of statins were statistically associated with AKI development (Table 4). The logistic regression model had an area under curve of 86.56% and the Hosmer-Lemeshow goodness-of-fit test resulted in *p* = 0.24. Based on this model, a one-unit increase in the serum HBA1c level increased the risk of AKI development by 44%, and patients with proteinuria were 12 times more likely to develop AKI.

Regardless of diabetes status, patients who developed AKI had significantly higher mortality rates compared with patients who did not develop AKI (*p* = 0.02). Moreover, there was a significant positive association between the severity of AKI and mortality (*p* = 0.01). There was also a marginally significant association between AKI development and invasive mechanical ventilation (*p* = 0.06); also, the need for invasive mechanical ventilation significantly differed across the stages of AKI (*p* = 0.003) (Table 5).

## 4. Discussion

In this retrospective cohort study, among 254 patients hospitalized for COVID-19 (127 with diabetes and 127 without diabetes), 58 patients (22.8%) developed AKI during hospitalization; patients with diabetes were more likely to develop AKI and also experienced more severe AKI compared with patients without diabetes. Based on the published literature, the rate of AKI varies widely among patients with COVID-19, ranging from 0% to 37% [6, 7, 17, 18]. The discrepancies in the reported AKI rates may be likely due to variations in the frequency of serum creatinine level measurements and in the definition of AKI across studies. Another possible explanation for the wide range of AKI prevalence might be differences in patient characteristics and disease severity. In our study, patients were recruited from a tertiary care hospital in Tehran, Iran, a center that generally admits patients with more severe COVID-19. Reports from the US and Europe also found a prevalence of 20-40% for AKI development in COVID-19 patients [4, 7, 19], whereas initial reports from China found that lower percentages of patients with COVID-19 had AKI [20–22]. For example, Wang et al. found that no patients developed AKI during hospitalization; however, that study probably included COVID-19 patients with mild disease as reflected by their mortality rate (6.0%) [17]. Diabetes mellitus, older age, and severe disease are independent risk factors for AKI development in ARDS secondary to non-COVID-19 pneumonia. In addition, the severity of AKI in these patients is positively associated with older age, higher BMI, and diabetes mellitus [23].

Multivariate analysis showed that in patients with diabetes, invasive mechanical ventilation, HBA1c level, proteinuria (any degree), history of cardiovascular disease, and use of statins were independent predictors of AKI development; patients who required invasive mechanical ventilation during hospitalization were approximately 11.6 times at greater risk of developing AKI. Previous retrospective studies also found a significant association between invasive mechanical ventilation and AKI development in patients with COVID-19 [7, 24]. Hirsch et al. found that 90% of patients who developed AKI had the need for invasive mechanical ventilation and that AKI developed in temporal association with respiratory failure [7]. No study, however, has yet described the risk factors associated with AKI development in the diabetic population with COVID-19. Generally, lung and kidney injuries are strongly interconnected; not only is ARDS associated with greater risk of AKI development but also patients with AKI are prone to respiratory failure and pulmonary complications [25]. As mentioned earlier, our study showed that proteinuria was an independent risk factor for AKI development among patients with diabetes and COVID-19; this finding supports previous studies underlining the strict relationship between albumin excretion rate and cardiorenal outcomes in diabetic nephropathy [26–28]. Furthermore, while no association was previously found between HBA1c level and mortality in patients with coexistent diabetes and COVID-19 [29, 30], this study showed that higher HBA1c levels put patients with diabetes at greater risk of developing AKI during hospital stay.

Besides AKI, kidney involvement manifested as proteinuria and hematuria in 55% and 38% of patients. As expected, patients with diabetes had higher rates of proteinuria, but no significant association was observed between hematuria and diabetes. Relatively similar percentages were reported for proteinuria and hematuria (43.9% and 26.7%, respectively) in another cohort study that included 701 patients with COVID-19 [21]. In addition, in a retrospective study performed on 333 hospitalized patients with COVID-19 in Wuhan, China, 75.4% had renal involvement, characterized by proteinuria, hematuria, and/or AKI [3].

In our study, the mortality rate among all patients was about 19% by the end of follow-up. Despite the higher percentage of patients requiring invasive mechanical ventilation, the longer in-hospital stay, and the markedly higher CT severity scores in patients with diabetes, no significant difference was observed between patients with and without diabetes in terms of mortality rate and COVID-19-related complications, such as septic shock and multiple organ failure. Nevertheless, patients who developed AKI, a finding that was more frequent in the diabetes group, had significantly higher rates of mortality as compared to those who did not experience AKI; about one-third of patients who developed AKI had a fatal outcome. Also, higher stages of AKI were associated with increased risk of death. The mortality rate of AKI in patients with SARS and MERS is greatly higher than that observed in this study for patients with COVID-19 [31]. Several other studies in patients with COVID-19 demonstrated higher mortality rates in those who developed AKI at some point during hospitalization [8, 32, 33]. In a study by Cheng and colleagues, Kaplan-Meier analysis revealed a greater risk of death in patients with COVID-19 who had kidney disease, which was defined as increased baseline serum creatinine, baseline BUN, proteinuria, hematuria, and AKI [21].

Recently, there has been a lot of speculation over the use of ACEI/ARB in patients with COVID-19 [34–36]. In the present study, although the use of ACEI/ARB in patients with diabetes was significantly associated with AKI by univariate analysis, no statistically significant association was found after adjustment for potential confounders. Similarly, no relationship was observed between AKI and treatment with renal-angiotensin-aldosterone–inhibiting medications among patients admitted with COVID-19 in the US [7]. However, a study conducted in France showed that the chronic use of ACEI/ARB was independently associated with AKI stage ≥ 1 in patients with severe COVID-19 [37].

Several underlying mechanisms have been proposed for kidney involvement associated with COVID-19. Direct virus entry into the kidneys, specifically proximal tubule cells and podocytes that express ACE2 and TMPRSS2, is one of the potential mechanisms of SARS-CoV-2-induced kidney injury [38]. This is supported by the presence of SARS-CoV-2 in the urine swabs of infected patients as well as the observation of viral particles on histopathological examination [5, 39, 40]. Hypovolemia at admission, right/left-sided heart failure, systemic inflammation and cytokine production, nephrotoxic drugs, and high positive end-expiratory pressure are other indirect mechanisms that may contribute to kidney involvement after infection with SARS-CoV-2 [4, 5, 41].

We acknowledge several limitations of this study. First, due to the unavailability and inaccuracy of preadmission laboratory data, serum creatinine values at admission were considered as baseline kidney function, which might not reflect true preadmission baseline renal function. Second, due to limitations in collecting 24-hour urine samples at the height of the pandemic, proteinuria was measured by using a semiquantitative method in this study. Third, there was missing data for urine tests, which indicates that the percentage of patients with proteinuria and/or hematuria may have been underestimated in this study.

In conclusion, the results of this study showed that AKI develops in a considerable percentage of patients with COVID-19, especially in those with diabetes, and is significantly associated with mortality. Among patients with diabetes, invasive mechanical ventilation, HBA1c level, proteinuria, cardiovascular disease, and use of statins were independent risk factors for AKI development.

## Figures and Tables

**Table 1:** Baseline characteristics of patients with COVID-19, by diabetes status.

Variable	Total	Diabetes	Nondiabetes	*p* value
Age (yrs)	65.7 (±12.5)	66.4 (±12.5)	65.0 (±12.5)	0.33
Sex				
Male	142 (55.9)	71 (55.9)	71 (55.9)	0.55
Female	112 (44.1)	56 (44.1)	56 (44.1)	
Respiratory rate (breaths/min)	18 (17-20)	18 (18-20)	18 (16-20)	0.02
Pulse rate (bpm)	88 (±15)	88 (±15)	88 (±15)	0.85
Oxygen saturation (%)	91 (87-94)	90 (85-92)	93 (90-95)	<0.001
SBP (mm/Hg)	120 (±17)	121 (±18)	119 (±15)	0.41
DBP (mm/Hg)	77 (±10)	78 (±10)	76 (±10)	0.44
Body mass index (kg/m^2^)	26.1 (24.0-29.2)	26.7 (25.0-30.8)	25.7 (23.4-27.6)	0.001
Positive RT-PCR assay	194 (76.4)	103 (81.1)	91 (71.7)	0.19
HbA1c (%)		8.6 (7.5-10.5)		
≤7	—	25 (20.7)	—	—
>7	—	96 (79.3)	—	—
Duration of diabetes				
New-onset	—	10 (7.9)	—	—
<5 years	—	23 (18.1)	—	—
5-10 years	—	38 (29.9)	—	—
10-15 years	—	24 (18.9)	—	—
>15 years	—	32 (25.2)	—	—
Presenting symptoms				
Cough	163 (64.6)	89 (70.1)	74 (58.3)	0.04
Productive	34 (20.2)	16 (18.0)	18 (24.3)	0.34
Nonproductive	129 (79.8)	73 (82.0)	56 (75.7)	
Chills	62 (24.4)	33 (26.0)	29 (22.8)	0.33
Fever	109 (42.9)	53 (41.7)	56 (44.1)	0.40
Dyspnea	152 (59.8)	83 (65.4)	69 (54.3)	0.05
Chest pain	29 (11.4)	14 (11.0)	15 (11.8)	0.51
Fatigue	92 (36.2)	49 (38.6)	43 (33.9)	0.26
Nausea/vomiting	44 (17.3)	26 (20.5)	18 (14.2)	0.12
Abdominal pain	16 (6.3)	7 (5.5)	9 (7.1)	0.40
Diarrhea	16 (6.3)	11 (8.7)	5 (3.9)	0.10
Anorexia	43 (16.9)	21 (16.5)	22 (17.3)	0.50
Headache	20 (7.9)	10 (7.9)	10 (7.9)	0.59
Loss of consciousness	16 (6.3)	11 (8.7)	5 (3.9)	0.10
Seizure	5 (2.0)	4 (3.1)	1 (0.8)	0.19
Myalgia	74 (29.1)	36 (28.3)	38 (29.9)	0.45
Comorbidities				
Smoking				
Current smoker	9 (3.5)	6 (4.7)	3 (2.4)	0.55
Ex-smoker	5 (2.0)	2 (1.6)	3 (2.4)	
Nonsmoker	240 (94.5)	119 (93.7)	121 (95.3)	
COPD	7 (2.8)	4 (3.1)	3 (2.4)	0.50
Hypertension	109 (42.9)	68 (53.5)	41 (32.3)	<0.001
Cerebrovascular disease	21 (8.3)	16 (12.6)	5 (3.9)	0.01
Malignancy	11 (4.3)	4 (3.1)	7 (5.5)	0.27
Immunodeficiency	5 (2.0)	4 (3.1)	1 (0.8)	0.19
Chronic kidney disease	21 (8.3)	16 (12.6)	5 (3.9)	0.01
Cardiovascular disease	64 (25.2)	39 (30.7)	25 (19.7)	0.03
Thyroid disease	12 (4.7)	6 (4.7)	6 (4.7)	0.62
Outcomes				
CT severity score	10 (7-14)	11 (8-14)	10 (7-13)	0.02
Clinical outcome				
Death	48 (18.9)	29 (22.8)	19 (15.0)	0.11
Recovery	206 (81.1)	98 (77.2)	108 (85.0)	
Invasive mechanical ventilation				
Yes	27 (10.6)	19 (15.0)	8 (6.3)	0.03
No	227 (89.4)	108 (85.0)	119 (93.7)	
Septic shock	21 (8.3)	13 (10.2)	8 (6.3)	0.18
Multiple organ failure	25 (9.8)	15 (11.8)	10 (7.9)	0.20
Length of hospitalization (days)	6 (3-10)	7 (5-12)	5 (2-9)	<0.001
Laboratory data				
Leukocyte (×10^3^/*μ*L)	6.1 (±3.3)	6.8 (±4.6)	6.4 (±7.6)	0.07
Lymphocyte (/*μ*L)	1204 (818-1797)	1209 (790-1795)	1194 (876-1846)	0.98
≤1500	161 (63.4)	80 (63.0)	81 (63.8)	0.96
Neutrophil (/*μ*L)	4417 (3289-7127)	4624 (3704-7544)	4137 (2912-6192)	0.04
Hemoglobin (g/dL)	12.5 (±2.0)	12.3 (±2.0)	12.7 (±2.0)	0.05
Platelet (×10^3^/*μ*L)	200 (±86)	208 (±92)	191 (±80)	0.13
ESR (mm/h)	47.9 (±26.1)	47.9 (±27.7)	47.9 (±24.5)	0.90
LDH (U/L)	483 (±552)	335 (±259)	427 (±382)	0.17
CRP (mg/L)	53.0 (±45.3)	64.5 (±61.9)	54.6 (±65.1)	0.66
D-dimer (ng/mL)	564 (±191)	514 (±1673)	576 (±1565)	0.85
Procalcitonin, ng/mL	0.23 (±0.37)	0.30 (±0.36)	0.28 (±1.11)	0.85
CPK (U/L)	123 (±160)	147 (±197)	114 (±126)	0.67
Albumin (g/dL)	3.8 (±0.6)	3.8 (±0.5)	3.9 (±0.6)	0.05
AST (U/L)	46 (± 24)	32 (± 16)	33 (± 27)	0.83
ALT (U/L)	23 (±18)	27 (± 11)	25 (± 25)	0.39
Creatinine (mg/dL)				
Admission	1.8 (1.4-2.4)	1.9 (1.4-2.5)	1.7 (1.4-2.2)	0.45
Peak	2.1 (1.5-3.3)	2.3 (1.5-3.5)	1.9 (1.6-2.7)	0.16
Discharge	1.6 (1.2-2.6)	1.7 (1.2-2.8)	1.5 (1.2-1.9)	0.16
Blood urea (mg/dL)	39.0 (27.2-60.6)	42.8 (29.0-65.5)	36.6 (26.5-49.8)	0.01
GFR (at admission) (mL/min/1.73 m^2^)	53.08 (38.62-66.14)	52.33 (34.76-65.14)	54.74 (44.17-67.08)	0.14

Data are presented as mean (± SD), median (IQR), and *n* (%). SBP: systolic blood pressure; DBP: diastolic blood pressure; RT-PCR: reverse transcriptase polymerase chain reaction; COPD: chronic obstructive pulmonary disease; ESR: erythrocyte sedimentation rate; LDH: lactate dehydrogenase; CRP: C-reactive protein; CPK: creatine phosphokinase; GFR: glomerular filtration rate; AST: aspartate aminotransferase; ALT: alanine aminotransferase.

**Table 2:** Medications used by patients during hospitalization.

Medication	Total	Diabetes	Nondiabetes	*p* value
Statins	66 (26.0)	43 (33.9)	23 (18.1)	0.004
ACEI/ARB	79 (31.1)	54 (42.5)	25 (19.7)	<0.001
Diuretics	24 (9.4)	15 (11.8)	9 (7.1)	0.20
CCB	20 (7.9)	12 (9.4)	8 (6.3)	0.35
Clopidogrel	15 (5.9)	12 (9.4)	3 (2.4)	0.02
Aspirin (ASA)	27 (10.6)	15 (11.8)	12 (9.4)	0.54
Warfarin	4 (1.6)	4 (3.1)	—	0.12
Heparin	252 (99.2)	125 (98.4)	127 (100.0)	1.00
Enoxaparin	2 (0.8)	2 (1.6)	—	0.50

ACEI/ARB: angiotensin-converting enzyme inhibitor/angiotensin II receptor blocker; CCB: calcium channel blocker.

**Table 3:** Kidney involvement among study subjects, by diabetes status.

Variable	Total	Diabetes	Nondiabetes	*p* value
AKI	58 (22.8)	36 (28.3)	22 (17.3)	0.04
Stage 1	22 (37.9)	9 (25.0)	13 (59.1)	0.03
Stage 2	21 (36.2)	15 (41.7)	6 (27.3)	
Stage 3	15 (25.9)	12 (33.3)	3 (13.6)	
Urine protein				
Negative/trace	18 (45.0)	8 (30.8)	10 (71.4)	0.01
Positive	22 (55.0)	18 (69.2)	4 (28.6)	
1 +	8 (36.4)	7 (38.9)	1 (25.0)	
2+	4 (18.2)	2 (11.1)	2 (50.0)	
≥3+	10 (45.4)	9 (50.0)	1 (25.0)	
Hematuria	16 (38.1)	10 (38.5)	6 (37.5)	0.95
Urine specific gravity	1.024 (± 0.006)	1.023 (± 0.007)	1.025 (± 0.004)	0.59
Need for RRT after AKI development	8 (13.8)	7 (19.4)	1 (4.5)	0.07

Data are presented as mean (± SD), median (IQR), and *n* (%). AKI: acute kidney injury; RRT: renal replacement therapy.

**Table 4:** Univariate and multivariate analyses of covariates associated with AKI in COVID-19 patients with diabetes.

Variable	Unadjusted OR	95% CI	*p* value	Adjusted OR	95% CI	*p* value
Age	1.01	0.98-1.04	0.51	0.98	0.93-1.03	0.47
Male sex	0.63	0.28-1.40	0.26	1.56	0.44-5.47	0.49
Body mass index	1.03	0.96-1.11	0.40	0.99	0.89-1.10	0.82
Cardiovascular disease	2.81	1.25-6.32	0.01	4.38	1.11-17.32	0.04^∗^
Hypertension	1.31	0.60-2.86	0.50	0.55	0.15-2.02	0.37
Chronic obstructive pulmonary disease	0.84	0.08-8.33	0.88	0.04	0.01-1.67	0.09
Statins	2.24	1.01-4.96	0.05	5.46	1.12-26.57	0.04^∗^
ACEI/ARB	2.10	0.96-4.58	0.06	1.53	0.38-6.18	0.55
Diuretics	3.43	1.14-10.31	0.03	1.78	0.28-11.48	0.54
Calcium channel blocker	1.94	0.57-6.55	0.29	6.67	0.86-52.04	0.07
Invasive mechanical ventilation	4.15	1.22-14.10	0.02	11.57	1.89-70.93	0.01^∗^
CT severity score	1.09	0.99-1.19	0.08	1.08	0.96-1.21	0.18
Hematuria	12.71	2.55-63.39	0.002	7.55	0.49-115.40	0.15
Proteinuria	7.08	2.41-20.85	<0.001	12.23	1.81-82.78	0.01^∗^
HBA1c	1.24	1.04-1.49	0.02	1.44	1.10-1.90	0.01^∗^

^∗^ *p* values are significant at the 0.05 level. AKI: acute kidney injury; OR: odds ratio; CI: confidence interval; ACEI/ARB: angiotensin-converting enzyme inhibitor/angiotensin II receptor blocker; CT: computed tomography; HBA1c: hemoglobin A1c.

**Table 5:** Clinical outcomes of patients, by AKI status and AKI stage.

Variable	AKI				No AKI	*p* value^a^	*p* value^b^
	Total	Stage 1	Stage 2	Stage 3			
Final outcome							
Recovery	41 (70.7)	17 (77.3)	18 (85.7)	6 (40.0)	165 (84.2)	0.02	0.01
Death	17 (29.3)	5 (22.7)	3 (14.3)	9 (60.0)	31 (15.8)		
Invasive mechanical ventilation							
No	48 (82.8)	21 (95.5)	19 (90.5)	8 (53.3)	179 (91.3)	0.06	0.003
Yes	10 (17.2)	1 (4.5)	2 (9.5)	7 (46.7)	17 (8.7)		

^a^Between the AKI and no AKI groups. ^b^Between the stages of AKI. AKI: acute kidney injury.

## Data Availability

Data supporting the findings of this study are available from the corresponding author upon request.

## References

[1] World Health Organization (WHO) Coronavirus disease (COVID-2019) situation report -199. https://www.who.int/emergencies/diseases/novel-coronavirus-2019/situation-reports.

[2] Cummings M. J., Baldwin M. R., Abrams D. (2020). Epidemiology, clinical course, and outcomes of critically ill adults with COVID-19 in New York City: a prospective cohort study. *The Lancet*.

[3] Pei G., Zhang Z., Peng J. (2020). Renal involvement and early prognosis in patients with COVID-19 pneumonia. *Journal of the American Society of Nephrology*.

[4] Ronco C., Reis T., Husain-Syed F. (2020). Management of acute kidney injury in patients with COVID-19. *The Lancet Respiratory Medicine*.

[5] Gabarre P., Dumas G., Dupont T., Darmon M., Azoulay E., Zafrani L. (2020). Acute kidney injury in critically ill patients with COVID-19. *Intensive Care Medicine*.

[6] Richardson S., Hirsch J. S., Narasimhan M. (2020). Presenting characteristics, comorbidities, and outcomes among 5700 patients hospitalized with COVID-19 in the New York City area. *Journal of the American Medical Association*.

[7] Hirsch J. S., Ng J. H., Ross D. W. (2020). Acute kidney injury in patients hospitalized with COVID-19. *Kidney International*.

[8] Xu S., Fu L., Fei J. (2020). Acute kidney injury at early stage as a negative prognostic indicator of patients with COVID-19: a hospital-based retrospective analysis. *medRxiv*.

[9] Goldman M. D. (2003). Lung dysfunction in diabetes. *Diabetes Care*.

[10] Knapp S. (2013). Diabetes and infection: is there a link?--a mini-review. *Gerontology*.

[11] Zhou F., Yu T., du R. (2020). Clinical course and risk factors for mortality of adult inpatients with COVID-19 in Wuhan, China: a retrospective cohort study. *Lancet*.

[12] Haseli S., Khalili N., Bakhshayeshkaram M., Sanei Taheri M., Moharramzad Y. (2020). Lobar distribution of COVID-19 pneumonia based on chest computed tomography findings; a retrospective study. *Archives of Academic Emergency Medicine*.

[13] Abrishami A., Khalili N., Dalili N. (2020). Clinical and radiologic characteristics of COVID-19 in patients with CKD. *Iranian journal of kidney diseases.*.

[14] Katulanda P., Dissanayake H. A., Ranathunga I. (2020). Prevention and management of COVID-19 among patients with diabetes: an appraisal of the literature. *Diabetologia*.

[15] Kellum J. A., Lameire N., Aspelin P. (2012). Kidney disease: improving global outcomes (KDIGO) acute kidney injury work group. KDIGO clinical practice guideline for acute kidney injury. *Kidney International Supplements.*.

[16] Levey A. S., Bosch J. P., Lewis J. B., Greene T., Rogers N., Roth D. (1999). A more accurate method to estimate glomerular filtration rate from serum creatinine: a new prediction equation. Modification of Diet in Renal Disease Study Group. *Annals of Internal Medicine*.

[17] Wang L., Li X., Chen H. (2020). Coronavirus disease 19 infection does not result in acute kidney injury: an analysis of 116 hospitalized patients from Wuhan, China. *American Journal of Nephrology*.

[18] Ng J. J., Luo Y., Phua K., Choong A. M. T. L. (2020). Acute kidney injury in hospitalized patients with coronavirus disease 2019 (COVID-19): a meta-analysis. *J Infect*.

[19] GECOVID working group, Russo E., Esposito P. (2020). Kidney disease and all-cause mortality in patients with COVID-19 hospitalized in Genoa, Northern Italy. *Journal of Nephrology*.

[20] Guan W. J., Ni Z. Y., Hu Y. (2020). clinical characteristics of coronavirus disease 2019 in China. *New England Journal of Medicine*.

[21] Cheng Y., Luo R., Wang K. (2020). Kidney disease is associated with in-hospital death of patients with COVID-19. *Kidney International*.

[22] Wu C., Chen X., Cai Y. (2020). Risk factors associated with acute respiratory distress syndrome and death in patients with coronavirus disease 2019 pneumonia in Wuhan, China. *JAMA Internal Medicine*.

[23] Panitchote A., Mehkri O., Hastings A. (2019). Factors associated with acute kidney injury in acute respiratory distress syndrome. *Annals of Intensive*.

[24] Fominskiy E. V., Scandroglio A. M., Monti G. (2020). Prevalence, characteristics, risk factors, and outcomes of invasively ventilated COVID-19 patients with acute kidney injury and renal replacement therapy. *Blood Purification*.

[25] Joannidis M., Forni L. G., Klein S. J. (2020). Lung–kidney interactions in critically ill patients: consensus report of the acute disease quality initiative (ADQI) 21 workgroup. *Intensive Care Medicine*.

[26] Minutolo R., Sasso F. C., Chiodini P. (2006). Management of cardiovascular risk factors in advanced type 2 diabetic nephropathy: a comparative analysis in nephrology, diabetology and primary care settings. *Journal of Hypertension*.

[27] Sasso F. C., Chiodini P., Carbonara O. (2012). High cardiovascular risk in patients with type 2 diabetic nephropathy: the predictive role of albuminuria and glomerular filtration rate. The NID-2 Prospective Cohort Study. *Nephrology Dialysis Transplantation*.

[28] Minutolo R., Gabbai F. B., Provenzano M. (2018). Cardiorenal prognosis by residual proteinuria level in diabetic chronic kidney disease: pooled analysis of four cohort studies. *Nephrology Dialysis Transplantation*.

[29] for the CORONADO investigators, Cariou B., Hadjadj S. (2020). Phenotypic characteristics and prognosis of inpatients with COVID-19 and diabetes: the CORONADO study. *Diabetologia*.

[30] Raoufi M., Khalili S., Mansouri M., Mahdavi A., Khalili N. (2020). Well-controlled vs poorly-controlled diabetes in patients with COVID-19: are there any differences in outcomes and imaging findings?. *Diabetes Research and Clinical Practice*.

[31] Chen Y.-T., Shao S.-C., Lai E. C.-C., Hung M.-J., Chen Y.-C. (2020). Mortality rate of acute kidney injury in SARS, MERS, and COVID-19 infection: a systematic review and meta-analysis. *Critical Care*.

[32] Lim M. A., Pranata R., Huang I., Yonas E., Soeroto A. Y., Supriyadi R. (2020). Multiorgan failure with emphasis on acute kidney injury and severity of COVID-19: systematic review and meta-analysis. *Canadian Journal of Kidney Health and Disease*.

[33] Ali H., Daoud A., Mohamed M. M. (2020). Survival rate in acute kidney injury superimposed COVID-19 patients: a systematic review and meta-analysis. *Renal Failure*.

[34] Fang L., Karakiulakis G., Roth M. (2020). Are patients with hypertension and diabetes mellitus at increased risk for COVID-19 infection?. *The Lancet Respiratory Medicine*.

[35] Esler M., Esler D. (2020). Can angiotensin receptor-blocking drugs perhaps be harmful in the COVID-19 pandemic?. *Journal of Hypertension*.

[36] Vaduganathan M., Vardeny O., Michel T., McMurray J. J. V., Pfeffer M. A., Solomon S. D. (2020). Renin-angiotensin-aldosterone system inhibitors in patients with Covid-19. *New England Journal of Medicine*.

[37] Oussalah A., Gleye S., Clerc Urmes I. (2020). Long-term ACE inhibitor/ARB use is associated with severe renal dysfunction and acute kidney injury in patients with severe COVID-19: results from a referral center cohort in the northeast of France. *Clinical Infectious Diseases*.

[38] Pan X. W., Xu D., Zhang H., Zhou W., Wang L. H., Cui X. G. (2020). Identification of a potential mechanism of acute kidney injury during the COVID-19 outbreak: a study based on single-cell transcriptome analysis. *Intensive Care Medicine*.

[39] Su H., Yang M., Wan C. (2020). Renal histopathological analysis of 26 postmortem findings of patients with COVID-19 in China. *Kidney International*.

[40] Diao B., Feng Z., Wang C. (2020). Human kidney is a target for novel severe acute respiratory syndrome coronavirus 2 (SARS-CoV-2) infection. *MedRxiv.*.

[41] Ronco C., Reis T. (2020). Kidney involvement in COVID-19 and rationale for extracorporeal therapies. *Nature Reviews. Nephrology*.

